# Mutational Pattern Induced by 5-Fluorouracil and Oxaliplatin in the Gut Microbiome

**DOI:** 10.3389/fmicb.2022.841458

**Published:** 2022-04-28

**Authors:** Li Wan, Hexin Li, Gaoyuan Sun, Lili Zhang, Hongtao Xu, Fei Su, Shunmin He, Fei Xiao

**Affiliations:** ^1^Clinical Biobank, Beijing Hospital, National Center of Gerontology, Institute of Geriatric Medicine, Chinese Academy of Medical Sciences, Beijing, China; ^2^The Key Laboratory of Geriatrics, Beijing Hospital, National Center of Gerontology, National Health Commission, Institute of Geriatric Medicine, Beijing Institute of Geriatrics, Chinese Academy of Medical Sciences, Beijing, China; ^3^Department of Laboratory Medicine, Beijing Hospital, National Center of Gerontology, Institute of Geriatric Medicine, Chinese Academy of Medical Sciences, Beijing, China; ^4^Key Laboratory of RNA Biology, Center for Big Data Research in Health, Institute of Biophysics, Chinese Academy of Sciences, Beijing, China

**Keywords:** chemotherapeutic agent, gut microbiota, microbes, gene mutation, genomic variation analysis

## Abstract

Chemotherapeutic agents, such as 5-fluorouracil (5-FU) and oxaliplatin (Oxi), can not only kill the cancer cell but also influence the proliferation of gut microbiota; however, the interaction between these drugs and gut microbiota remains poorly understood. In this study, we developed a powerful framework for taxonomy composition and genomic variation analysis to investigate the mutagenesis effect and proliferation influence of chemotherapeutic agents, such as 5-FU and Oxi, on gut microbiota and the interaction between these drugs and gut microbiota during chemotherapy. Using the gut microbiome data, we detected 1.45 million variations among the chemotherapy groups and found the drugs significantly affected mutation signatures of gut microbiota. Oxi notably increased transversion rate, whereas 5-FU reduced the rate. Traits related to cell division and nutrient mobilization showed evidence of strong selection pressure from chemotherapeutic agents. In addition, drug-associated bacteriome shift patterns and functional alterations were found: the metabolism changes in the 5-FU group implied that gut microbiota could provide additional nicotinamide adenine dinucleotide (NAD^+^) to inhibit cancer cell autophagy; in the Oxi group, the ribosome and lysine biosynthesis genes were obviously enriched. Our study provides a blueprint for characterizing the role of microbes and drug–microbe interaction in the gut microbiota response to chemotherapy.

## Introduction

Chemotherapeutics have long been used to treat a variety of human tumors (Kemp and Kwon, 2021). As one of the commonly used chemotherapy protocols, the FOLFOX regimen contains different chemotherapeutic agents [e.g., 5-fluorouracil (5-FU) and oxaliplatin (Oxi)] with a broad range of cytotoxicity (de Gramont et al., 2000). These drugs can effectively inhibit DNA replication of cancer cells via different mechanisms of action. For example, 5-FU produces FdUMP, which directly inhibits thymidylate synthase and thus causes thymine-less cell death (Pinedo and Peters, 1988). In addition, the products of 5-FU may be converted into FUTP or FdUTP, leading to RNA or DNA damage, respectively. An understanding of the interaction of chemotherapeutics and gut microbial populations will probably trigger applications toward improving the effectiveness of chemotherapy (Yuan et al., 2018).

Chemotherapeutic agents may affect gut microbial communities by disrupting the homeostatic balance among resident microorganisms; meanwhile, they can accelerate the microbe evolution at a molecular level (Montassier et al., 2015; Baym et al., 2016; Hong et al., 2019). Many recent studies have explored the interaction between microbiota and anticancer drugs along with interventions aimed at shaping microbiota to optimize drug efficacy and reduce side effects (Cheng et al., 2020). However, previous studies mainly focused on the taxonomic characterization and functional compositions of different cohorts at the genus or species level (Lloyd-Price et al., 2017). Variations in taxonomic abundance as well as functions encoded by these gut microbiota have been described in the cohorts of inflammatory bowel disease (IBD) (Halfvarson et al., 2017), type 2 diabetes (T2D) (Qin et al., 2012), hypertension (Li et al., 2017), and liver cirrhosis (Qin et al., 2014). However, genomic variations within species, which lead to their phenotypic diversity and adaptations to chemotherapeutic agents, have been studied in only a few taxa (Morowitz et al., 2011). For example, in the common gut commensal bacteria *Escherichia coli* (Bagel et al., 1999), only few point mutations can confer clinically relevant antibiotic resistance, and the natural variation in a single gene can lead to pathogenic adaptation.

Given the importance of the gut microbiota in human health and a growing number of studies reporting associations between gut microbiota and diseases (Hauser et al., 2015), a better understanding of the interaction between gut microbiota and chemotherapeutic drugs during chemotherapy helps to improve the efficacy of the anticancer chemotherapeutic agents. We recruited 37 cancer patients and collected prechemotherapy and postchemotherapy stool samples to study the taxonomic composition, metabolic capacity, and molecular evolution during the chemotherapy. In order to avoid the bias caused by distinct diet, regional, and genetic differences, we collected healthy samples from well-known studies about Han Chinese with the standard clinical assessments and same sequence platform to build the basic of microbiome structure and mutational pattern. We analyzed our cohort and stool samples from T2D (Qin et al., 2012), liver cirrhosis (Qin et al., 2014), and hypertension cohorts (Li et al., 2017) with the same bioinformatics pipeline and parameters. Our goal was to build a power framework for metagenomics analysis to gather basic knowledge on the genomic variation landscape and taxonomic abundance shift in gut microbiota metagenomes during chemotherapy.

## Materials and Methods

### Cohort Recruitment and Sample Collection

Participants were recruited in the Oncology Department of Beijing Hospital, Beijing, China, in 2018. This study was approved by the Beijing Hospital Ethics Committee. Written informed consent was obtained from all of the participants. The exclusion criteria included the following: with a history of IBDs; having been exposed to probiotics, prebiotics, or broad-spectrum antibiotics within 30 days; or having received nasal-tube feeding or parenteral nutrition in the month prior to initiation of the study. Detailed information about all patients is listed in Supplementary Table 1. We collected two fecal samples from each participant. A fecal sample was collected on hospital inpatient admission (day 0), prior to administration of chemotherapy, and 30 days later immediately prior to chemotherapy (day 30), respectively. For each fecal sample, 1 g of stool was collected into a sterile tube and then subsequently stored at –80°C for molecular analysis. Total genomic DNA was extracted from 200 to 500 mg of fecal sample using a QIAamp DNA Stool Mini kit (Qiagen, Germany) according to the manufacturer’s instructions. The concentration of the extracted DNA was measured by a NanoDrop2000 (Thermo Fisher Scientific, United States), and then the DNA was stored at –80°C for the next studies.

### 16S Ribosomal RNA Sequencing and Bioinformatics Analysis

To develop the 16S rRNA amplicon libraries, the V3–V4 region of 16S rRNA gene was polymerase chain reaction–amplified with the primers 341F (5′-CCTAYGGGRBGCASCAG-3′) and 806R (5′-GGACTACNNGGGTATCTAAT-3′), modified by adding barcodes for multiplexing (Caporaso et al., 2012). Pooled amplicons were paired-end sequenced [PE 2 × 250 base pairs (bp)] by using an Illumina HiSeq 2500 platform according to the manufacturer’s protocol. Paired-end reads were merged and quality filtered using FLASH and QIIME (Bokulich et al., 2013). Chimeras were detected and removed against the Gold reference database using the UCHIME algorithm (Edgar, 2013). Sequences with ≥ 97% similarity were assigned to the same OTUs. For each representative sequence, the GreenGenes Database was used based on the RDP classifier algorithm to annotate. α Diversity analysis including Shannon and Chao1 was calculated with QIIME. Phylogenetic β diversity distances, including unweighted and weighted UniFrac distances, were measured using QIIME. Principal component analysis was performed to visualize by ggplot2 package in R software (version 3.5.0). Further, diversity analyses, such as Adonis and Anosim, were performed by running a workflow on QIIME.

### Shotgun Metagenomics Sequencing Library Construction

For shotgun metagenomics sequencing, adaptive focused acoustics (Covaris) was used to shear a standard volume of 50 μL of the extracted DNA. DNA libraries were prepared using Illumina TruSeq Sample Preparation Kit (Illumina, United States) according to the manufacturer’s protocol. The DNA libraries were quantified using an Agilent Bioanalyzer 2100 (Agilent Technologies, United States). Paired-end sequencing (PE 2 × 150 bp) was performed on successful DNA libraries using an Illumina HiSeq X-Ten instrument at the Annoroad Genome Biotech in Beijing. Finally, we obtained 4.27–8.9 GB of raw data for each sample (average, 5.98 GB; Supplementary Table 2).

### Metagenomics Bioinformatics Analysis

Concatenated and human DNA sequences were removed using KneadData with default parameter.^1^ The remaining high-quality reads were assembled with IDBA-UD (version 2.04, parameters: -pre_correction -min_contig 200) (Peng et al., 2012). We mapped the clean reads against scaffolds using Bowtie2 (version 2.3.4.2) (Langmead and Salzberg, 2012). Genes (minimum length of 100 nucleotides) were predicted on contigs longer than 500 bp and annotated using Prokka (Parameters: -metagenome) (Seemann, 2014). The gene abundance was determined by using a method similar to RPKM (reads per kilo bases per million reads) used for quantifying gene expression from RNA sequencing data. In brief, high-quality reads were counted with HTSeq-count (Anders et al., 2015). For each gene, G_i_, the number of reads that aligned to it divided by the length of the gene was calculated as NG_i_, and the relative abundance, RNG_i_, of each gene in each sample (n genes) was computed using the following formula:


$${\text{RNG}}_{i}={\text{NG}}_{i}/\sum_{i=1}^{n}\text{NG}i$$

Species-level quantitative taxonomic profiling was performed using MetaPhlAn2 (version 9760413b180f) on the KneadData-filtered reads (Truong et al., 2015). Community composition was calculated with MetaPhlan2 using the default settings. Taxonomic profiles including bacteria, archaea, microbial eukaryotes, and viruses were inferred by MetaPhlAn2 using the 1M unique clade-specific marker genes identified from 17,000 reference genomes (13,500 bacterial and archaeal, 3,500 viral, and 110 eukaryotic).

### Functional Annotation

Two different methods were used for functional alteration and pathway composition analysis. Pathway-level composition was calculated with HUMANn2 using the UniRef90 database with default settings, which was followed by further statistical analysis and visualization in STAMP (Parks et al., 2014). In order to obtain the detailed information of each metabolic pathway, all genes in our genomes were aligned to the Kyoto Encyclopedia of Genes and Genomes (KEGG) database using DIAMOND (version 0.7.9.58, parameter: -k 50 -sensitive -e 0.00001) and KOBAS (Xie et al., 2011). Each protein was assigned to the KEGG Orthology (KO) families by the highest-scoring annotated hits containing at least one HSP scoring over 60 bits. The abundance of KO/module was calculated using the methods as mentioned previously. For KO enrichment analysis, the significant enriched KEGG pathway was defined as the adjusted *p*-value (< 0.05) of the hypergeometric test as the previous study (Jiang et al., 2016).

### Generation of a Reference Genome Set

We followed the previous method to build the reference genomes to study the genomic variation of gut microbiota (Schloissnig et al., 2013). Approximately 3,934 prokaryotic genomes were downloaded from NCBI^2^ on August 1, 2018. A set of 40 universal single copy marker genes was identified in these genomes using HMM profiles made for each marker gene from the corresponding orthologous group from the eggnog database (Ciccarelli et al., 2006; Sorek et al., 2007). For each marker gene, pairwise DNA sequence identities among all genomes were calculated using BLASTn (version 2.2.25, parameter: *e* < 10^–5^ -F F) (Altschul et al., 1990). For each genome pair, the median identity of all marker genes was used as a proxy for average nucleotide identity (ANI) between the two genomes. Using an operational 95% ANI recommended for identifying species, we generated 929 clusters of genomes. In order to select a reference genome from each cluster, high-quality reads from a subset of shotgun metagenomes (bgi-BGI-06A, bgi-DLF001, bgi-DOF002 from T2B cohort; nHF611710, nHF411719 from HTN; HD-2, HD-31 from liver; 1-1, 4-1, 5-1 from this study) were mapped to the 3934 genomes using Bowtie2 with the options “–very-fast.” Then, the genome with the highest read coverage was selected, resulting in a set of 958 reference genomes, each likely representing a unique species (Supplementary Table 7).

### Mapping to Reference Genomes and Variant Calling

Illumina reads from 718 fecal samples, including the T2D, liver, and hypertension cohorts were quality controlled using KneadData. KneadData-filtered reads were mapped to reference genomes using Bowite2 (version 2.3.4.2) with default parameter. The number of reads mapping to reference genomes was counted and normalized by the genome size in order to obtain quantitative relative abundances of each genome in every sample.

To improve the accuracy of genomic variation, we modified the protocol of the previous study (Schloissnig et al., 2013). The SAMtools/BCFtools suite was used for calling high-quality InDel supported by more than five reads and at least one read on each strand (Li et al., 2009). We then performed local realignment of the aligned reads with those high-quality InDels using the Genome Analysis Toolkit (GATK) (McKenna et al., 2010) to minimize the mismatch bases. GATK’s HaplotypeCaller was used to call variants with default parameter. BreakDancer was used to detect the structural variation with at least three reads supporting the event (Fan et al., 2014).

### Positive Selection Analysis

Coding DNA sequences (CDSs) were annotated based on their protein family membership using KOBAS (Xie et al., 2011). With MUSCLE (Edgar, 2004), a multiple sequence alignment (MSA) of the protein sequences was created using each KO. Based on the MSA and the CDS nucleotide sequences, a codon-based alignment was constructed for each KO module with PAL2NAL using default parameters (Suyama et al., 2006). We then used FastTree, a relaxed neighbor joining algorithm, to reconstruct a phylogenetic tree for each protein family from the obtained MSA (Price et al., 2009). We excluded low-confidence positions in the alignment with a large number of gaps with Gblocks (Castresana, 2000). Dn/Ds was calculated with PAML (Yang, 2007). A one-sided Fisher test was performed to identify protein families with a significant enrichment of Dn vs. Ds changes in comparison to the entire sample. The false discovery rate (FDR) was controlled using the Benjamini and Hochberg procedure and α set to 5%.

### *In vitro* Experiment to Validate the Mutational Pattern Shift

A filter-sterilized stock solution of 100 mM 5-FU, Oxi, and 5-FU + Oxi (1:1) (Sigma–Aldrich) was prepared in dimethyl sulfoxide and further diluted to 50, 75, and 100 mM. Stock solutions were further diluted (1:1,000) in culture medium for the experiments. Four mono-isolated gut microorganisms were obtained from the stools of recruited patients. Luria broth (LB) agar plates used for passaging were prepared by adding stock 5-FU, Oxi, and 5-FU + Oxi. Subsequently, cultures were transferred into fresh plates and allowed to grow 48 h for each batch. After 20 batches, single colony on each plate was then scraped off and transferred to Eppendorf tubes with liquid LB medium and cultured overnight at 37°C. Total DNA was then extracted using the QIAamp UCP Pathogen Mini Kit (Qiagen). The concentration of the extracted DNA was measured by a NanoDrop2000 (Thermo Fisher Scientific, United States). Illumina DNA sequence libraries were prepared as mentioned previously. JSpeciesWS was used to assign the taxonomy of each isolated strain (Richter et al., 2016). Reads were then mapped to the closest reference genomes with BWA, and variant calling was performed as mentioned previously.

### Statistical Analysis

The Wilcoxon signed-ranks test was performed to evaluate differences in richness and diversity for KEGG and microbial taxa at various taxonomic ranks. Mutation rate comparisons were conducted to determine statistical significance between two groups for incidences where differences were declared significant at *p* < 0.05 with the Student *t*-test.

### Data Availability

All sequences generated in this study have been available in the NCBI sequence read archive under the accession number PRJNA551354.

## Results

### Overall Composition of Gut Microbiota During Chemotherapy

**FIGURE 1:**
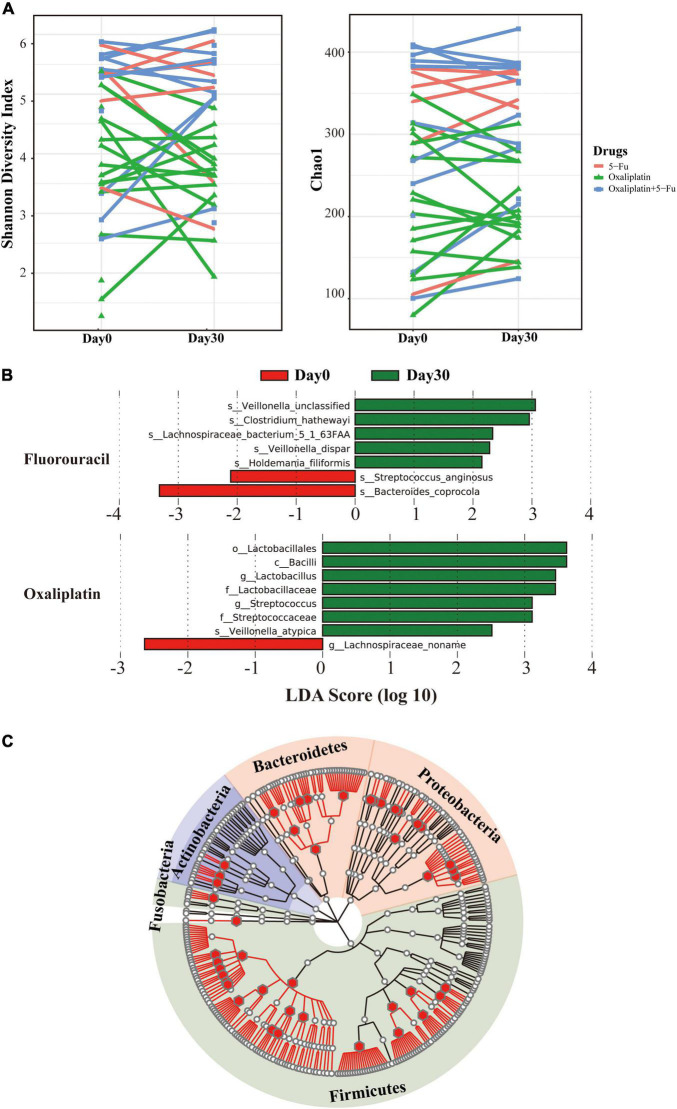
Gut microbial diversity before and after the chemotherapy. **(A)** Shannon diversity (left) and Chao (right) based on 16*S* rRNA sequencing. Different colors stand for drugs: red for 5-fluorouracil, green for oxaliplatin, and blue for 5-fluorouracil and oxaliplatin. The *y*-axis stands for the collection time. **(B)** Cladogram derived from LEfSe analysis of metagenomic sequences based on the shotgun sequencing comparing day 0 with day 30. Green shaded areas indicate microbe orders that more consistently describe the fecal microbiome from day 30; red shaded areas indicate microbe orders that more consistently describe from day 0. The prefixes “c,” “o,” “f,” “g,” and “s” represent the annotated level of class, order, family, genus, and species. **(C)** Comparison of 16*S* rRNA and metagenome abundances. The tree represents all taxonomically classified species from the shotgun metagenome survey as well as 16*S* rRNA sequence. The branches of the tree do not reflect evolutionary distances. The position of the dots in the tree corresponds to the taxonomic placement of the representative sequences in the NCBI taxonomy. Empty dots represent the phylotypes found in the shotgun metagenome classification; red dots were identified from both methods.

To characterize the diversity and composition of gut microbiome during the chemotherapy of the patients, we characterized the gut microbial composition of 68 fecal samples from 37 patients (six samples were excluded because of DNA extraction failure) using 16S rRNA gene sequencing. Detailed information about stool samples and patients is listed in Supplementary Tables 1, 2. This cohort consisted of 7 patients using 5-FU, 13 patients using 5-FU + Oxi, and 17 patients using Oxi. All the participants were from a cohort study in Beijing Hospital. Using the samples collected at the start of chemotherapy (day 0) and 30 days later (day 30), we traced the dynamic changes of gut microbial species by ecological α and β diversity measures. The chemotherapeutic drugs caused modest changes in the gut microbiome. Shannon diversity and Chao1 index were calculated to estimate the within-sample (α) diversity (Figure 1A), and there was no significant alteration pattern. Weighted UniFrac principal coordinate analysis was used to compare community phylogenetic composition among samples, and the results revealed a separation between days 0 and 30 along axis 1 (explaining 41.5% of the variation in the data, Supplementary Figure 1). β Diversity represented how much the community changed in comparison to the baseline (day 0) and was calculated with weighted UniFrac distance between days 0 and 30 (Supplementary Table 3). ANOSIM with permutations confirmed significant separation of samples (*p* = 0.02, *r* = 0.24). No significant differences in microbial communities were observed when the Oxi and 5-FU + Oxi groups were analyzed separately. Although the adjusted *p* (adjusted *p* = 0.06) was not significant, it indicated that there was a difference pattern in the microbial composition between days 0 and 30 with 5-FU. Next, we used linear discriminant analysis (LDA) effect size (LEfSe) to analyze the microbial communities of different chemotherapeutic drugs. For all day 30 samples, we identified gut microbiome signatures with a higher abundance of bacilli (specifically Streptococcaceae and Lactobacillaceae, Figure 1B). For 5-FU, seven species were discovered as biomarkers for separating gut microbiota between days 0 and 30 (Supplementary Figure 2). Five of these species were higher, and two were lower on day 0 than on day 30. For example, the abundances of *Bacteroides coprocola* and *Streptococcus anginosus* were lower on day 30 (*p* = 0.03, two-sided Wilcoxon signed-ranks test). Contrary to 5-FU, in the Oxi group, eight of these species were higher, and one was lower on day 0 than on day 30. No significant changes were observed in the 5-FU + Oxi group. To gain further insights into the interaction between chemotherapeutic drugs and gut microbiota, we used the same gut microbiota DNA preparations for independent Illumina shotgun sequencing. The yielded sequences belonged to 10 phyla and 381 species, including several DNA viruses (Supplementary Table 4 and Supplementary Figure 3). *Firmicutes* was the most abundant phylum, accounting for 40.9% ± 22.3% of the total reads, followed by Bacteroidetes (36.3% ± 23.7%) and Proteobacteria (15.7% ± 23.4%), similar to previous studies (Qin et al., 2014). Two commensal species including *Streptococcus salivarius* and *Ligilactobacillus salivarius* were significantly enriched after the chemotherapy (Supplementary Figure 4). Correlation tests between the abundance estimates for bacterial taxa at genus level were performed by using the two methods (Pearson correlation coefficient: 0.84; *p* < 2.2 × 10^–16^; Figure 1C and Supplementary Figure 5), highlighting a highly positive correlation between 16S rRNA and shotgun metagenomics sequencing.

### Functional Alterations in Gut Microbiota During the Chemotherapy

**FIGURE 2:**
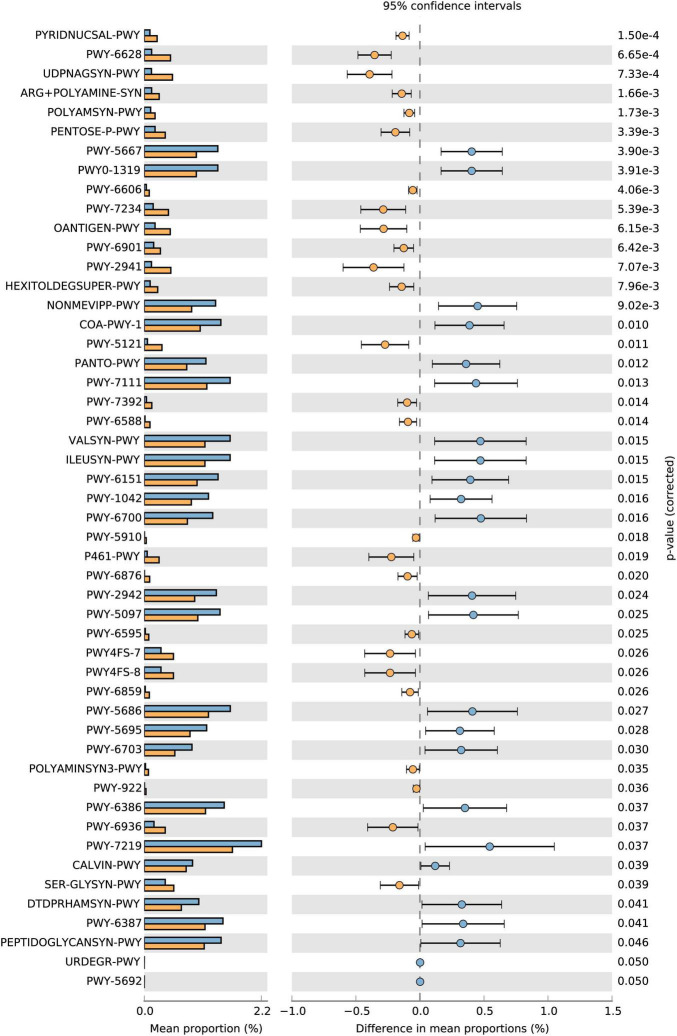
Extended error bar plots showing the abundance of pathways differing significantly between days 0 and 30 in the 5-FU group. Extended error bar plots showing the abundance of pathways differing significantly between days 0 and 30 in the 5-FU group. Corrected *p*-values are shown on the right. Adjusted *p* < 0.05 was considered significant. The yellow color stands for day 30 and blue for day 0.

To determine the functional alterations with the chemotherapeutic agents, we used the HUMAnN2/MinPath (Franzosa et al., 2018) and KEGG database (Kanehisa et al., 2017) to evaluate gut microbial functions across groups in our study cohort. According to the HUMAnN2/STAMP analysis of the metabolic function pathways (Parks et al., 2014), most differences occurred in carbohydrate metabolism and energy-related pathways in the 5-FU group (Figure 2). The microbiome from day 30 included more genes involved in nicotinamide adenine dinucleotide (NAD) salvage pathway (PYRIDNUCSAL-PWY) than that from day 0 in the 5-FU group (adjusted *p* = 1.4 × 10^–4^). A previous study suggested an active role for the NAD salvage pathway in modulating cancer cell viability via the replenishing of the NAD reservoir (Sharif et al., 2016), which is a novel strategy to protect the cell against DNA damage. Moreover, amino acid biosynthesis, such as L-phenylalanine and arginine, UDP-*N*-acetyl-D-glucosamine biosynthesis pathway were more active in the samples in the 5-FU group. In the other two groups, few pathways were found to be significantly different before and after chemotherapy (Supplementary Figure 6).

In order to deeply explore the functional role of the gut microbiota during the chemotherapy, all the genes were aligned to the KEGG database, and the abundance of KO groups and pathways for each sample were estimated using a method similar to RPKM (Hu et al., 2013). Approximately 277 KOs were significantly enriched among the three groups (*p* < 0.05, two-sided Wilcoxon rank sum test; Supplementary Table 5). Overrepresentation of three KEGG pathways involved in metabolism was observed in the 5-FU group with hypergeometric distribution, which is involved in the categories “galactose metabolism” and “phosphotransferase system (PTS)” (Supplementary Table 6). Enriched genes encoding PTS, which reflected increased representation of multiple carbohydrate transporters, were an important mechanism to confer resistance to selection pressure, such as antibiotic, as reported in the previous studies (Stogios et al., 2016). In the Oxi group, two KEGG pathways “ribosome” and “lysine biosynthesis” were significantly enriched. Although Oxi, a platinum-based drug, is generally considered to be DNA-damaging agents, recent studies have unexpectedly shown that Oxi did not alter DNA integrity but instead inhibited rDNA transcription, leading to p53 induction, most likely through the “impaired ribosome biogenesis checkpoint” (Pelletier et al., 2018).

### Genomic Variation in Intestinal Microorganisms

To enable comparative analyses in multiple metagenomes and to identify the mutation signatures in the different groups, we modified the previous method (Schloissnig et al., 2013) to identify single-nucleotide polymorphisms (SNPs), short insertions/deletions (InDels; range, 1–50 bp), and structural variations (>50 bp) in each sample. We used 3,934 prokaryotic genomes to generate a set of reference genomes (Supplementary Table 7) for the analysis of genomic variation in gut microbial species in 786 samples. Next, local realignment around high-quality InDels, which was a frequently used strategy in genomic variation analysis, was performed to correct mapping errors to improve the accuracy of variant calling (DePristo et al., 2011). We only considered variants with allele frequency larger than 1% and supported by more than five reads. To validate our SNP calling procedure, we used the approaches to calculate error rates in 40 essential single-copy marker genes, following the method mentioned previously (Schloissnig et al., 2013). False-positive rates were estimated at 3.6% (Supplementary Table 8).

We identified 1.45 million SNPs from 343 genomes (at least three SNPs for one genome), of which 1.38 million SNPs (95%) in 47 genomes (0.88% of the total 156 Mb) across 68 stool samples from 37 subjects (Supplementary Figure 7). We also identified 26,662 InDels and 56,820 structural variants. Subsequent analyses were restricted to SNPs because of their orders-of-magnitude higher count over other variation types. Examination of the SNP distributions of the protein coding genes of the selected 47 gut bacteria revealed that 4,189 genes with at least one non-synonymous mutation in the 47 microbe species had valid coverage (≥ 10 × depth) with sufficient prevalence (Supplementary Table 9). Among them, we identified 22 genes (0.5%) with significantly differentiated SNP densities between days 0 and 30 samples (Wilcoxon signed-ranks test, *p* < 0.05). Most of these genes (17/22) were found in *Bacteroides thetaiotaomicron* VPI-5482, the second most common human commensal bacteria, whose reference genome contains 4,779 protein-coding genes (Xu et al., 2003; Supplementary Table 10).

**FIGURE 3:**
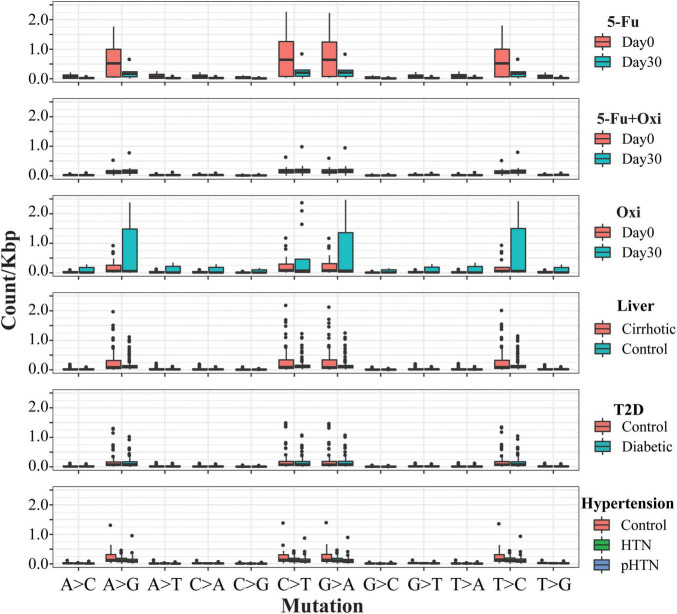
Mutational signatures found in all fecal samples. Each cohort is displayed according to the 12 substitutions. The different groups are displayed in different colors as the legends show. The mutation types are on the horizontal axes, whereas vertical axes depict the number of mutations detected in the kilobase attributed to a specific mutation type.

Then, we checked the substitution pattern shift between days 0 and 30. A large portion of base substitutions was attributable to C > T substitution, which ranged from 29.5 to 43.0% on day 0 and from 35.0 to 42.3% on day 30. Comparisons of the mutation rates in the other Chinese cohorts, including liver cirrhosis (Qin et al., 2014), hypertension (Li et al., 2017), and T2D (Qin et al., 2012; Figure 3 and Supplementary Table 11), showed a significantly reduced transversion (Student’s *t*-test, adjusted *p* = 0.0003) and transition rate (Student’s *t*-test, adjusted *p* = 0.01) in the 5-FU group (Supplementary Table 12). By contrast, we found an increased ratio of transversion (Student *t*-test, adjusted *p* = 5.4 × 10^–4^) and transition (Student’s *t*-test, adjusted *p* = 8.2 × 10^–7^) in the group of Oxi. The similar mutational pattern was also observed in the studies about the effect of antibiotic on the genome (Long et al., 2016). Thus, we conclude that genome-wide substitutions rate of gut microbiota were influenced by chemotherapeutic drugs. To directly test whether selection might have biased the mutation rate of different regions in the genomes, we examined the synonymous and non-synonymous mutation from the coding region from all cohorts. Non-synonymous/synonymous mutation ratio from day 30 was not significantly different from day 0 (Student’s *t*-test, *p* > 0.05 in all comparison), indicating that the vast majority of acquired amino acid–altering mutations was not selectively promoted by chemotherapeutic agents but simply accumulated in a neutral fashion.

In order to validate the shift of mutational pattern, we explored how stool-isolated microorganism mutation rates change *in vitro* when treated with 5-FU and Oxi. We exposed four gut species (two *E. coli* strains, *Citrobacter* sp. MGH104 and *Enterococcus faecium*) from four different patients, all of which were with significantly low growth rate limited by the chemotherapeutic agents, in different concentrations of 75 μM 5-FU and Oxi (Supplementary Figure 8). Most notably, the transition/transversion ratio of mutations covaried with agents concentrations (Supplementary Figure 9).

### Dn/Ds Across Gut Species and Individuals

**FIGURE 4:**
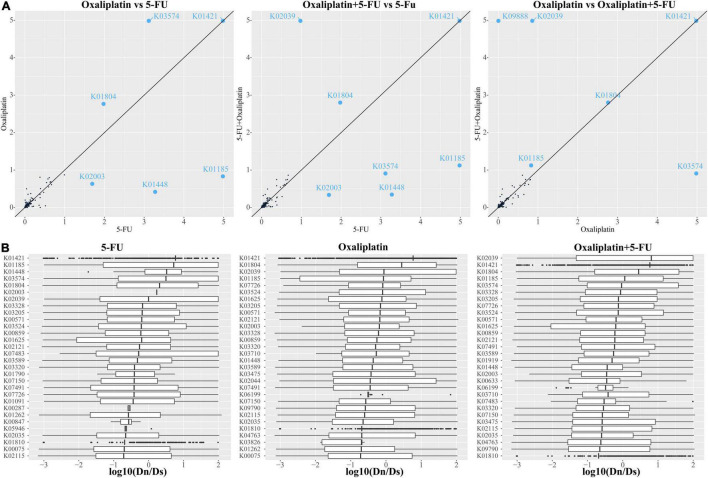
KEGG modules under positive selection in different chemotherapeutic drugs. **(A)** The correlation of average Dn/Ds ratio of all KO modules between the drugs. KO modules with average Dn/Ds ratio larger than 1 were marked by blue points. Maximum ratio was limited at 5. **(B)** Boxplot of top 20 ranked KO modules under positive selection with significantly increased Dn/Ds statistic, with sorted by their median log transformed Dn/Ds in descending order. The row stand for different KO modules.

To gain further insights on the molecular mechanisms driving the functional diversification of the gut microbiota, the gene families identified in the assembled metagenome were annotated based on the KEGG, and we calculated, for each KO, the ratio between the number of non-synonymous (Dn) and synonymous (Ds) changes, a proxy for evolutionary pressure (Supplementary Figure 10). Our analyses showed that the average Dn/Ds was 0.063, and the median was 0.016 from 939 KO (Supplementary Table 13 and Supplementary Figure 10). Approximately 17.9% (17.6% in the 5-FU group, 17.7% in the 5-FU + Oxi group, and 18.3% in the Oxi group) of the gene families had significantly higher Dn values and lower Ds values than the mean value calculated over all annotated sequences (one-sided Fisher test, FDR < 0.05), suggesting that they might be under positive selection (Supplementary Table 14 and Supplementary Figure 11). A closer investigation of these gene families revealed that positive selection signatures markedly characterized diverse proteins involved in the cell division, as well as proteins essential for amino acid biosynthesis (Figure 4 and Supplementary Figure 12). According to the abundance analysis of KO items between days 0 and 30, we found that genes were under significant positive selection pressure, such as *secY*, which is a key in the protein secretion system and important for critical cell functions, such as pathogens and virulence. In addition, as in the previous study (Bulgarelli et al., 2015), we also found the coding sequence of phage infection protein (*yhg*E) and the genes related to transport system were under positive selection. Thus, the genes under positive selection might have a key role in the interaction between bacteria and chemotherapeutic drugs and provide the bacteria additional survival advantage during the chemotherapy.

## Discussion

The human gut microbiota is highly complex and exists in a dynamic balance between symbiosis and pathogenesis, which can influence almost any aspect of host physiology (Sommer and Backhed, 2013). Growing evidences suggest that the gut microbiota not only plays a key role in carcinogenesis but also influences the efficacy and toxicity of anticancer therapy (Panebianco et al., 2018; Pothuraju et al., 2021). The microbiota modulates the host response to chemotherapy via numerous mechanisms, such as alteration of community structure and immune microenvironment. Furthermore, exploitation of the microbiota offers opportunities for the personalization of chemotherapeutic regimens and the development of novel therapies.

Chemotherapeutic agents, Oxi and 5-FU, exert their cytotoxic effect mostly through DNA damage. When DNA damage is caused by chemotherapeutic drugs, the microbiota composition also changes, which can further affect drug efficacy and overall health. It is very important to systematically explore the interaction between chemotherapeutic drugs and gut microbiota not only from microbiome composition (Yu et al., 2017; Hakim et al., 2018) but also genomic variation (Schloissnig et al., 2013; Zeevi et al., 2019). In this study, we first used two different methods (16*S* rRNA and shotgun metagenomics sequencing) to investigate the disruption of the intestinal microbiome in terms of taxonomic composition. Correlation tests between the abundance estimates for bacterial taxa showed that the two methods highlighted a highly positive correlation. According to the 16*S* rRNA sequence, we found that different chemotherapeutic agents had a distinct effect on the gut microbiota. 5-FU–associated bacteriome shifts included depletion of common health-associated commensals from the genera *Streptococcus* and *Bacteroides* and enrichment of Gram-negative bacteria such as *Clostridium hathewayi* and Lachnospiraceae bacterium. A previous study had shown that 5-FU could improve T cell–dependent antitumor immunity (Vincent et al., 2010), and *Clostridia* strains play a key role in enhancing regulatory T cell abundance and inducing important anti-inflammatory molecules, such as interleukin 10 (Atarashi et al., 2013). In contrast, Oxi-associated bacteriome showed different shift patterns including depletion of commensals from the genus Lachnospiraceae noname and enrichment of genus *Lactobacillus* and *Streptococcus*.

Subsequently, we used shotgun metagenomics with a quantitative metagenomic species approach to identify which species significantly changed during the chemotherapy with different agents. Two commensal species including *S. salivarius* and *L. salivarius* were significantly enriched after the chemotherapy. According to the previous studies, these two species appeared to be highly resistant to 5-FU (Vanlancker et al., 2016), which might be the main driver for a community dysbiosis. We hypothesized that if 5-FU was responsible for the dysbiosis changes seen during chemotherapy, then depleted taxa should be susceptible to the drug whereas the enriched species would be resistant to 5-FU. The resultant damage to the intestinal barrier increases the risk of colitis, bacterial translocation, and infection. Probiotic supplementation may have extra beneficial effects to correct dysbiosis of gut microbiota after the chemotherapy.

Concomitant with the alteration of gut microbial composition, we also observed a dysbiosis in bacterial gene functions, and different chemotherapeutic agents showed distinct influence patterns. Microbial metabolism may cause side effects severe enough to necessitate cessation of chemotherapy. The metagenome of 5-FU patients was enriched in genes associated with NAD salvage pathway. As NAD^+^ plays central roles in a variety of biological processes ranging from cellular metabolism and energy production in both human and microbiology, it was reasonable to hypothesize that gut microbiota could provide additional NAD^+^ to inhibit cancer cell autophagy and enhance survival of cancer cells (Sharif et al., 2016). By contrast, the metagenome of gut microbiota in Oxi patients was enriched in the genes associated with ribosome. Oxi, unlike cisplatin and carboplatin, kills cancer cells not only through the DNA-damage response, but also by inducing ribosome biogenesis stress (Bruno et al., 2017). In order to adapt the selection pressure from Oxi, only the species with a high copy number of the genes related to ribosome can survive during the chemotherapy similar to cancer cells, which may be why the genes involved with ribosomes were significantly enriched.

Next, in order to study the effects of DNA damage caused by chemotherapeutic agents, we collected the metagenomics sequencing data of 768 stool samples from 755 Chinese human individuals with the standard clinical assessments and reanalyzed the shift of mutational pattern with unified bioinformatics pipeline. Consistent with our expectation, we found obvious shift in the mutation signatures. A significantly reduced transversion rate (G:C- > T:A) was observed in the 5-FU group. In contrast, we found an increased ratio of the transversion in the Oxi group. In addition, the genome-wide substitution rates of gut microbiota were influenced by chemotherapeutic drugs without bias among different genomic regions. To further confirm that the shifts of mutation signatures were due to strong and diverse environmental selection, we analyzed the patterns of correlation between gene function and the Dn/Ds ratio across all the KO modules. The stable Dn/Ds ratios of most KEGG modules (80.1%) between days 0 and 30 suggest that the core microbiome was evolutionarily conserved. However, some modules, such as the genes involved in cell division, protein export, and phage-related, showed a high-level selection pressure. A previous study has shown that phage-related genes usually have fast mutation rate and are under positive selection (Petersen et al., 2007). However, the other genes, such as *zap*A and *mut*T, which are housekeeping genes involved in cell amplification and previously are thought to be with slow rate of amino acid evolution, have remarkably high Dn/Ds ratio and are under positive selection pressure. On the other hand, in order to be adaptive to the pressure of chemotherapeutic agents, bacteria take different strategies to obtain the survival advantage during the therapy, for example, by secreting an amount of protein to modify the host microenvironment with *Sec* system, which was a potential chemotherapeutic target to many human pathogens.

## Conclusion

We have described the disordered profiles of gut microbiota in cancer patients treated with different chemotherapeutic agents, explored the mutational pattern of different drugs, and provided a new clue for the interaction between drugs and microbe. Our findings suggest that a distinct intestinal microbiome pattern of gut dysbiosis during the chemotherapy is dominated by bacilli and a lack of other bacteria, and the potential restorative influence of probiotic supplements should be investigated in the chemotherapeutic research. Positive selection on the protein export system provides an additional survival advantage for gut microbiota and could be a potential therapy target for gut dysbiosis.

## Data Availability Statement

The datasets presented in this study can be found in online repositories. The names of the repository/repositories and accession number(s) can be found in the article/Supplementary Material.

## Ethics Statement

The studies involving human participants were reviewed and approved by the Beijing Hospital Ethics Committee. The patients/participants provided their written informed consent to participate in this study.

## Author Contributions

FX and SH conceived the study and designed the experiments. FS and LW performed the experiments. HL, GS, and HX made clinical samples validation. LZ, FS, HL, and GS collected clinical samples and analyzed the clinical data. FS, LZ, and SH analyzed the high throughput sequencing data. LW, FS, and FX wrote the manuscript. All authors read and approved the final manuscript.

## Conflict of Interest

The authors declare that the research was conducted in the absence of any commercial or financial relationships that could be construed as a potential conflict of interest.

## Publisher’s Note

All claims expressed in this article are solely those of the authors and do not necessarily represent those of their affiliated organizations, or those of the publisher, the editors and the reviewers. Any product that may be evaluated in this article, or claim that may be made by its manufacturer, is not guaranteed or endorsed by the publisher.

## Footnotes

1
http://huttenhower.sph.harvard.edu/kneaddata

2
ftp://ftp.ncbi.nlm.nih.gov/genomes/refseq/bacteria
