# Supplementary material for: Mutational Pattern Induced by 5-Fluorouracil and Oxaliplatin in the Gut Microbiome
Source: Front Microbiol. 2022 Apr 28;13:841458. doi: 10.3389/fmicb.2022.841458 (PMC9101311; doi:10.3389/fmicb.2022.841458)
Supplement: Supplementary file 1 [file Data_Sheet_1.PDF]

Supplementary Figures

Figure S1 Beta-diversity comparisons of the gut microbiomes of the stool samples collected at Day0 and Day30 during the chemotherapy.

Figure S2 Cladogram derived from LEfSe analysis of metagenomic sequences based on the shotgun sequencing compared Day0 with Day30.

Figure S3 Taxonomic profile of the gut microbiomes of the samples collected before and after chemotherapy with MetaPhlAn2.

Figure S4 Box plot shows the relative abundance of four species significantly different in the conditions.

Figure S5 Scatter plot of Pearson's correlation of 16S rRNA sequencing and shotgun metagenomics sequencing

Figure S6 Extended error bar plots showing the abundance of pathways differing significantly between before and after taking 5-FU+ Oxaliplatin and Oxaliplatin.

Figure S7 General information of genomic variations from all groups.

Figure S8 Growth curves of gut microbiota isolated from stool over hours 0 to 39.

Figure S9 Mutational signatures found in all *in vitro* samples

Figure S10 Distribution of Dn/Ds across different chemotherapeutic drugs.

Figure S11 Venn diagram of KEGG Ortholog groups significantly higher Dn values and lower Ds.

Figure S12 Boxplot of top 20 ranked KO modules under positive selection with significantly increased Dn/Ds statistic, with sorted by their median log transformed Dn/Ds in descending order.

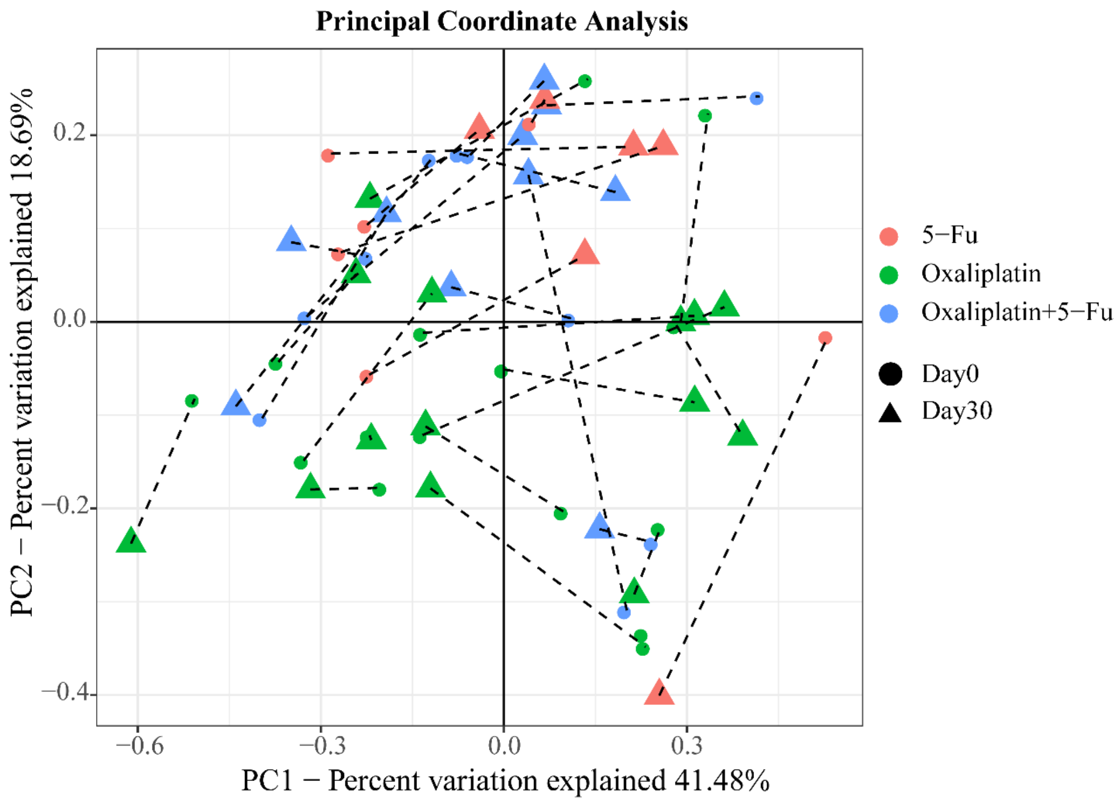

Supplementary Figure 1. Beta-diversity comparisons of the gut microbiomes of the faecal samples collected before (Day0) and after (Day30) chemotherapy.

Weighted UniFrac PCoA plot illustrates the samples from different patients at Day0 and Day30. Dots represent a sample collected at Day0 and triangles represent sample at Day30. Samples from same patient were connected with dash line. Different colors stand for chemotherapeutic drugs: red color for 5-FU, green for Oxaliplatin and blue for 5-FU + Oxaliplatin.

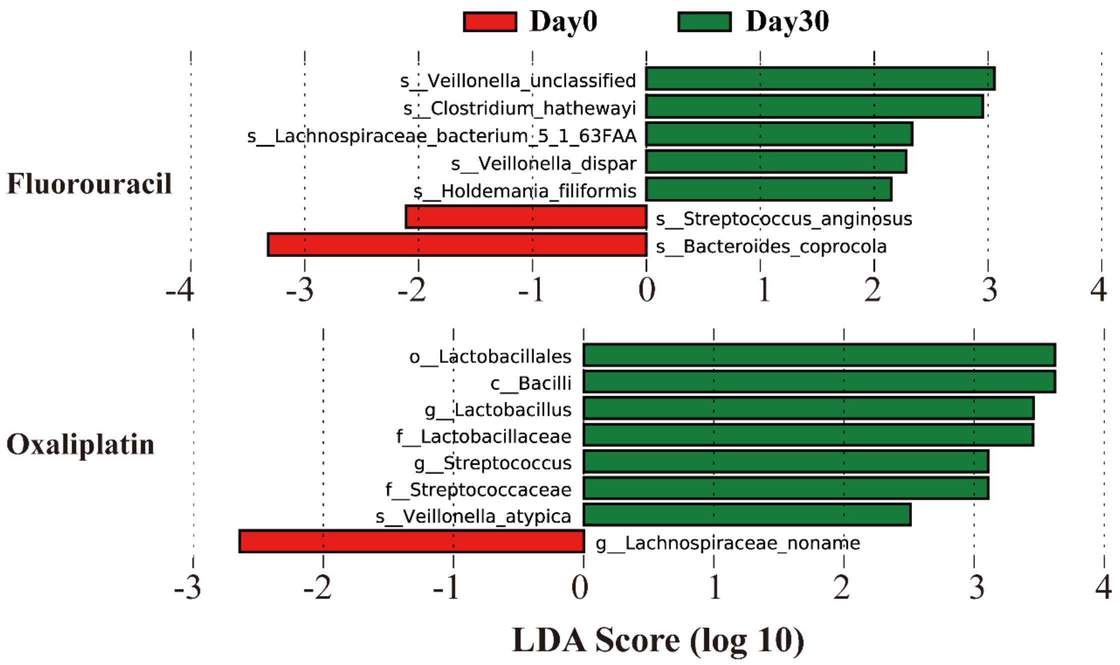

Supplementary Figure 2. Cladogram derived from LEfSe analysis of metagenomic sequences based on the shotgun sequencing compared Day0 with Day30.

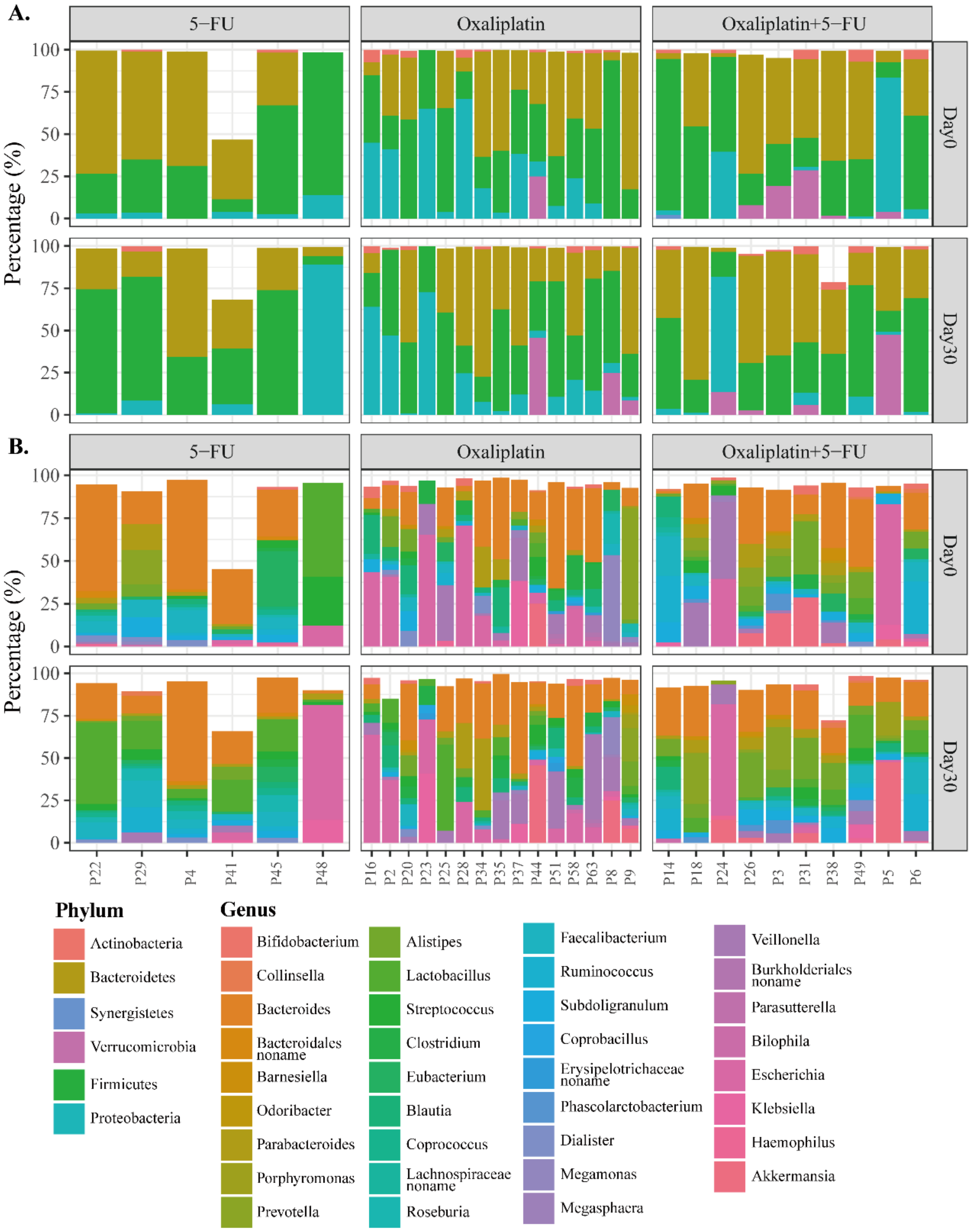

Supplementary Figure 3. Relative abundance of major bacteria (more than 1%) among the different groups.

Panel A is for phylum and panel B for genus. Different colors stand for the related phylotypes as show in the bottom.

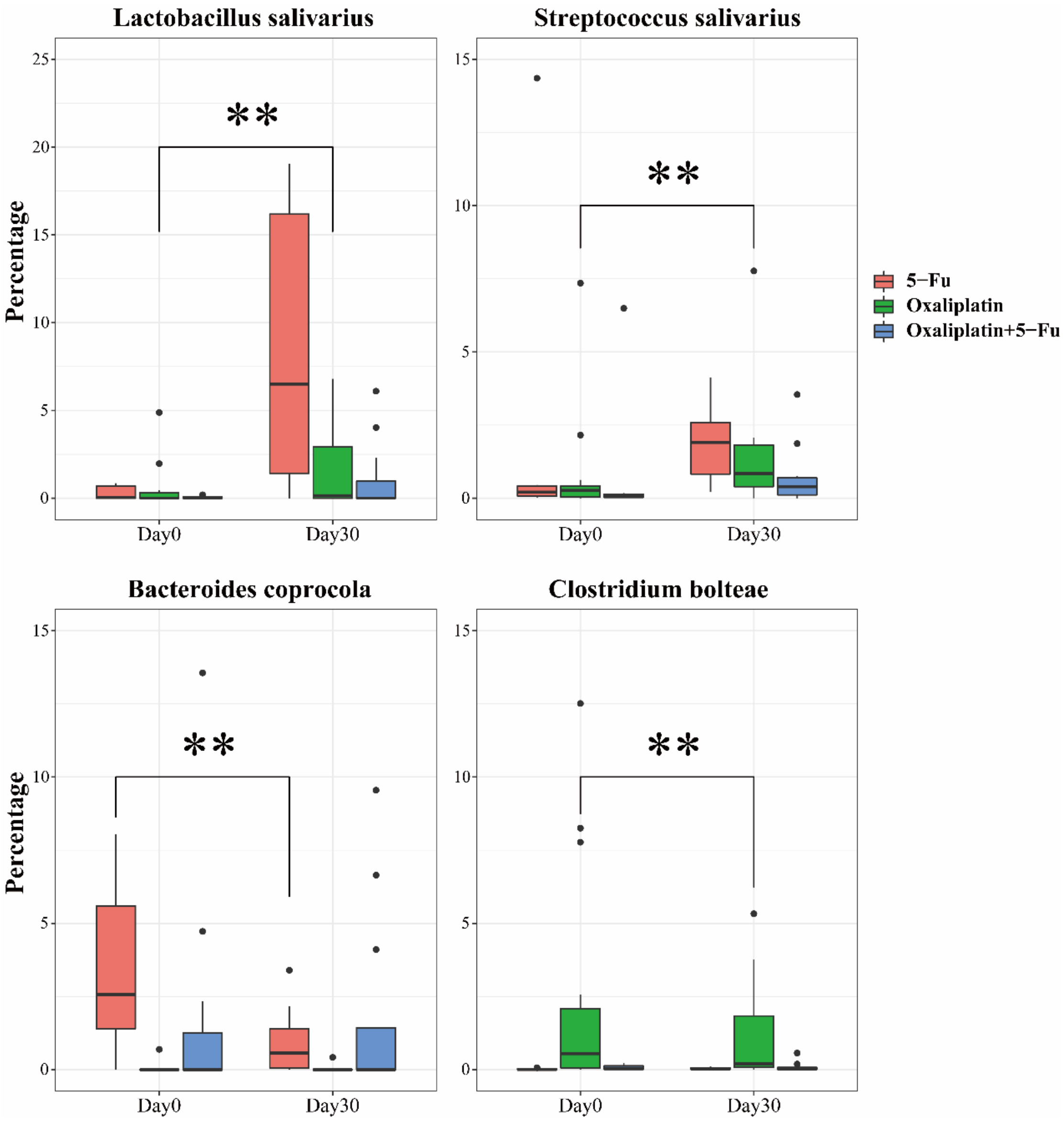

Supplementary Figure 4. The box plot shows the relative abundance of four species significantly different in the conditions.

Boxes represent the inter quartile ranges, lines inside the boxes denote medians, and points are outliers. Asterisk indicates the abundance with significantly difference before and after chemotherapy. Different colors stand for the different chemotherapeutic drugs, red for 5-Fluorouracil, green for Oxaliplatin and blue for 5-Fluorouracil + Oxaliplatin.

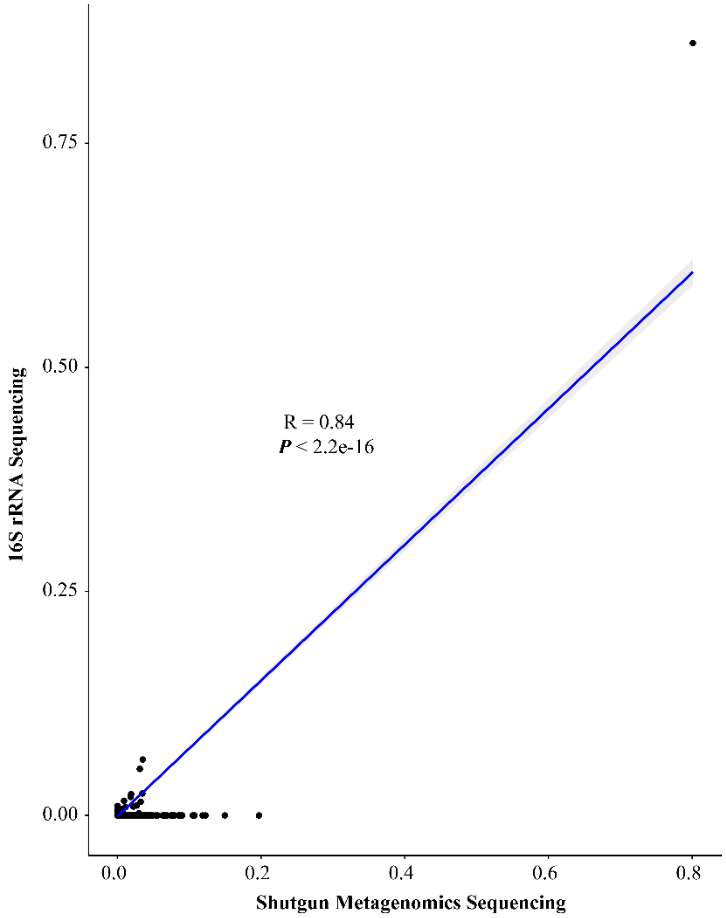

Supplementary Figure 5. Scatter plot of Pearson's correlation of 16S rRNA sequencing and shotgun metagenomics sequencing.

A.

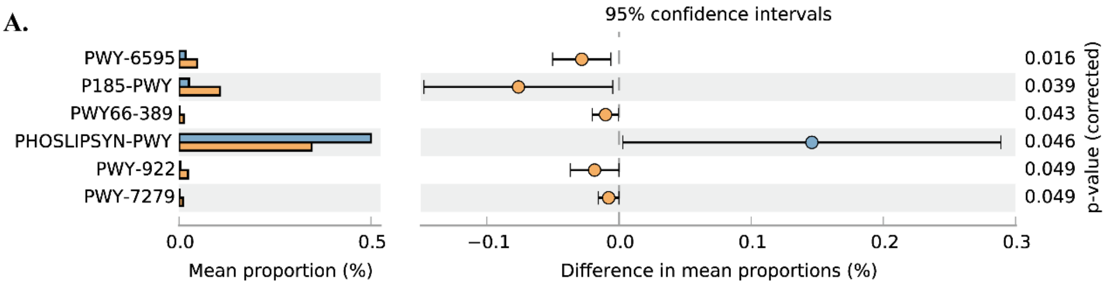

B.

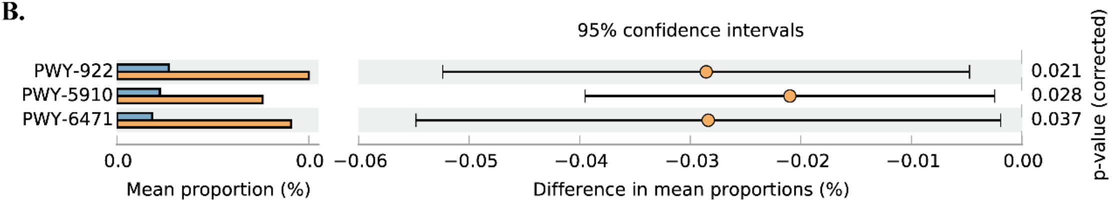

Supplementary Figure 6. Extended error bar plots showing the abundance of pathways differing significantly between before and after taking 5-FU + Oxaliplatin (Panel A) and Oxaliplatin (Panel B). Adjusted *P*-value < 0.05 was considered significant.

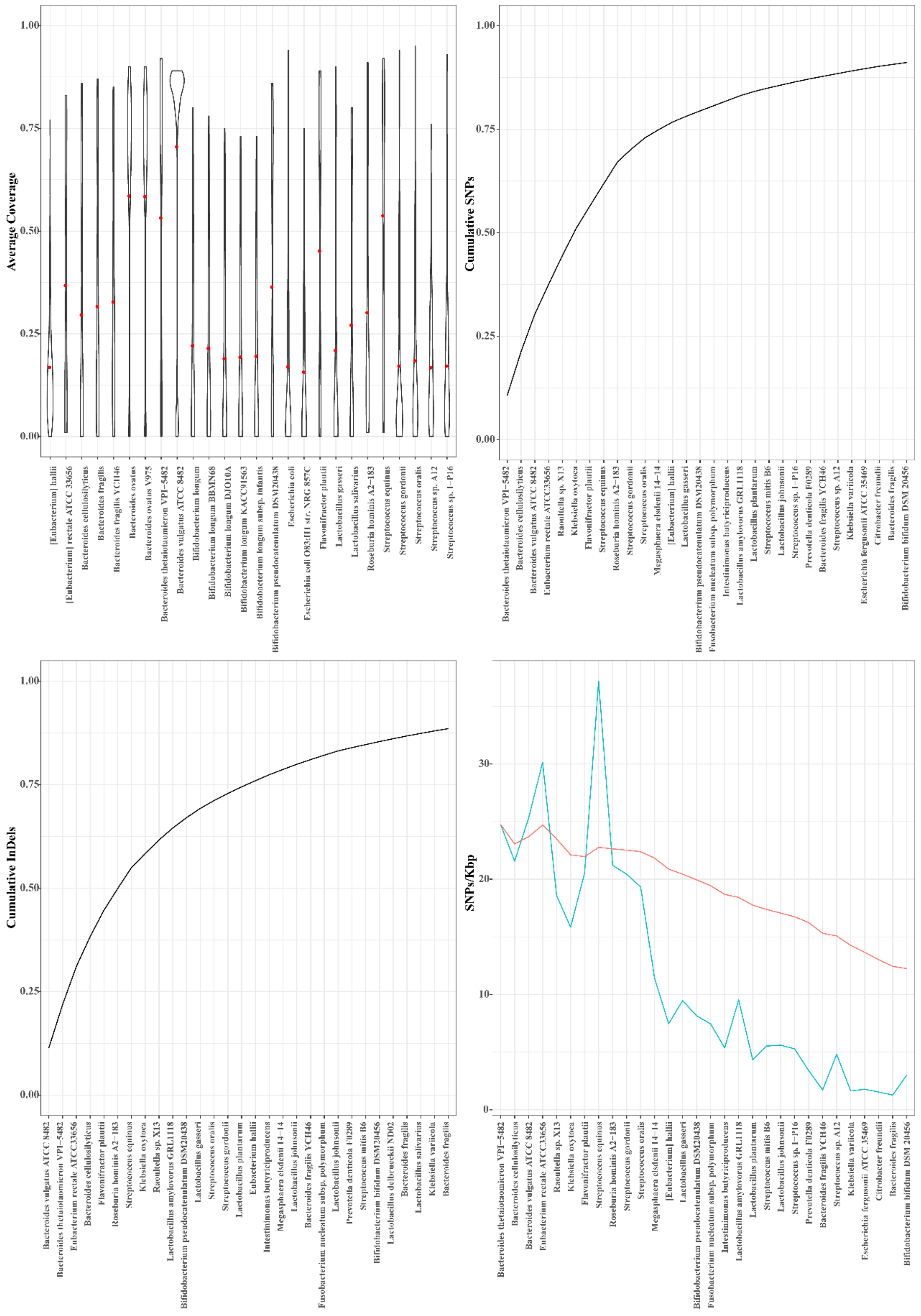

Supplementary Figure 7. General information of genomic variations from all groups.

A. Average coverage of top 25 species identified by shotgun metagenomics sequence.

The red dots show the median coverage across all samples and all samples were sorted by the total coverage.

B. Cumulated percentage of SNPs of top 30 species sorted by number of SNPs in each genomes

C. Cumulated percentage of InDels of top 30 species sorted by number of InDels in each genomes

D. Distribution of SNPs kb^{-1} in top 30 species. Red line stands for cumulated SNPs kb^{-1} and blue line for the SNPs kb^{-1} in each genome.

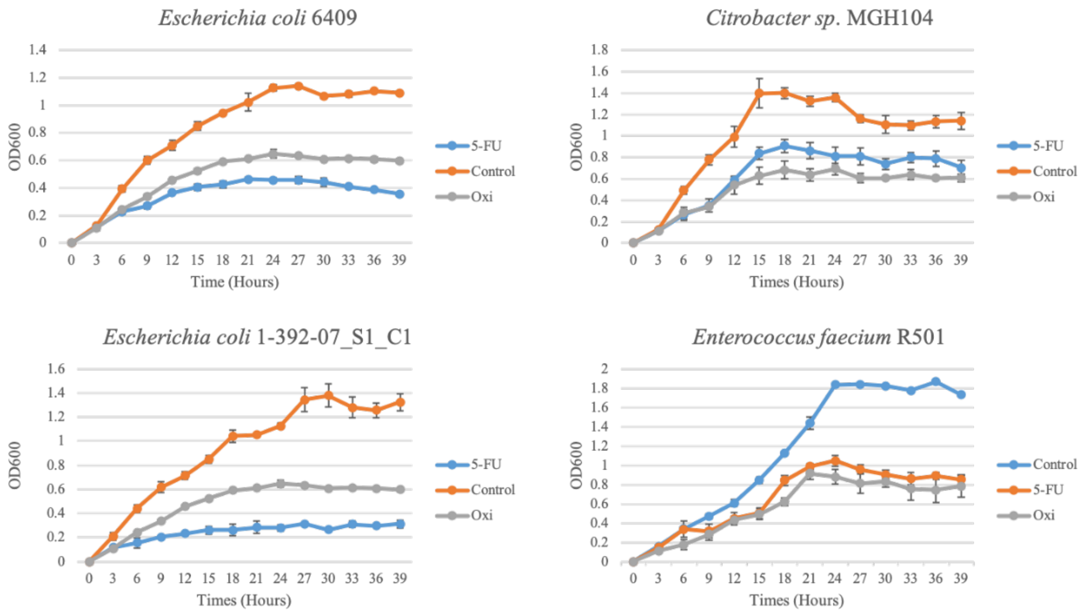

Supplementary Figure 8. Growth curves of gut microbiota isolated from stool over hours 0 to 39. Microbial growth was measured every 3 hours. Different colors stand for drugs.

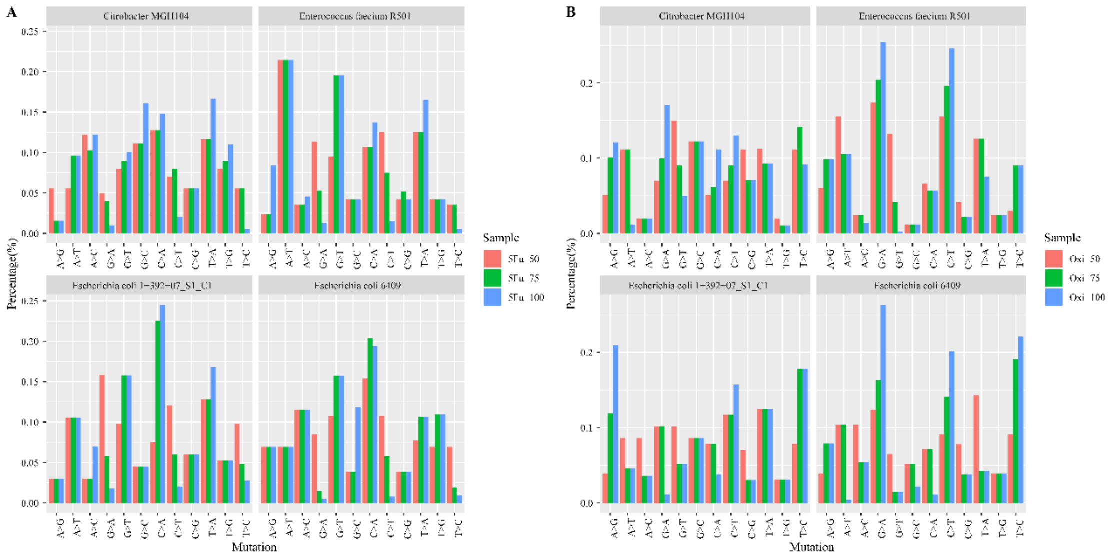

Supplementary Figure 9. Mutational signatures found in all *in vitro* samples

A is for the strains exposed to different 5-FU concentration and B for the strains exposed to Oxi. Different colors strand for agents concentrations, red is 50 µM, green is 75 µM and blue is 100 µM.

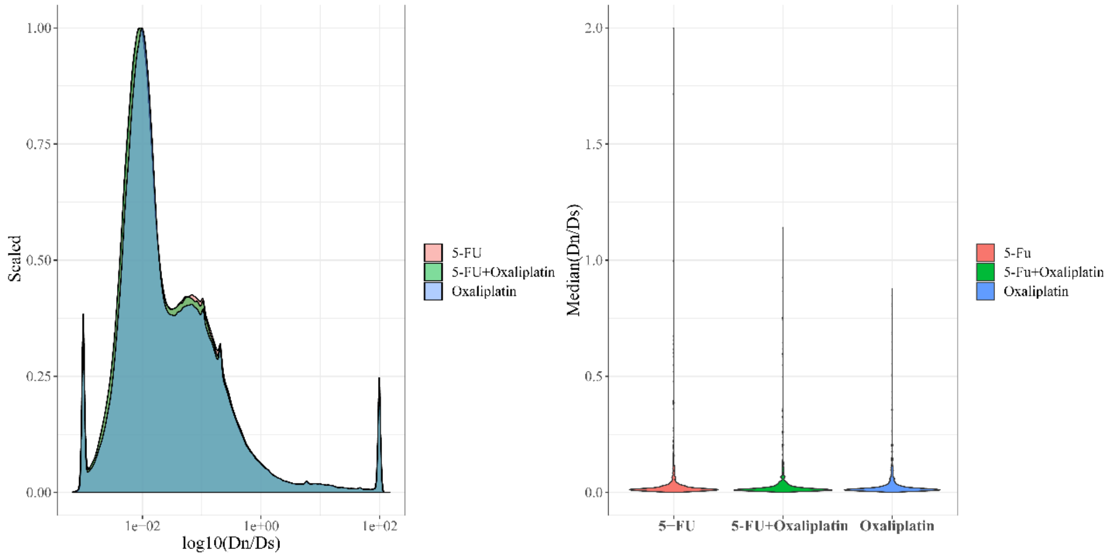

Supplementary Figure 10. Distribution of Dn/Ds across different chemotherapeutic drugs.

A. Density plot show the distribution of log transformed Dn/Ds;

B. Violin plots show the distribution of Median of Dn/Ds across different KEGG Ortholog groups. Boxes represent the inter quartile ranges, lines inside the boxes denote medians, and points are outliers. Different colors stands for various drugs as the legend.

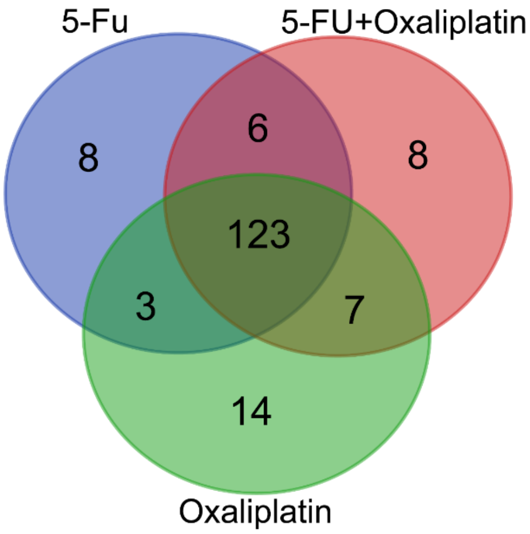

Supplementary Figure 11. Venn diagram of KEGG Ortholog groups significantly higher Dn values and lower Ds.

The red cycle stands for 5-FU+Oxaliplatin, the green cycle stands for Oxaliplatin and the blue cycle stands for the 5-FU.

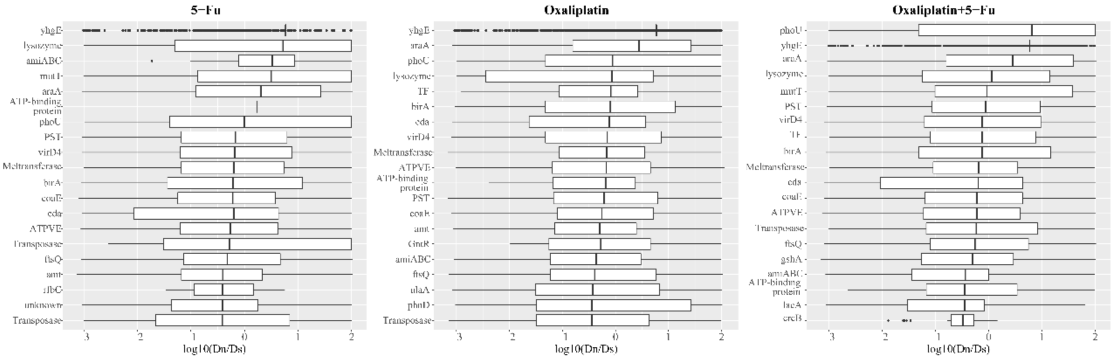

Supplementary Figure 12. Boxplot of top 20 ranked KO modules under positive selection with significantly increased Dn/Ds statistic, with sorted by their median log transformed Dn/Ds in descending order.
